# Postnatal growth of etiologically characterized preterm newborns according to gestational age at birth

**DOI:** 10.1038/s41390-024-03735-x

**Published:** 2024-11-28

**Authors:** Paola Roggero, Irina Ryumina, Robert B. Gunier, Adele Winsey, Stephen A. Rauch, Irma Alejandra Coronado Zarco, Shabina Ariff, Maria Albertina S. Rego, Constanza Soto Conti, Roseline Ochieng, Funda Tuzun, Jacqueline Asibey, Francesca Giuliani, Montserrat Izquierdo Renau, Chandrakala Bada Shekharappa, Alexandre Lapillonne, Gabriela Tavcioska, Leila Cheikh Ismail, Camilla Menis, Marina Markelova, Ricardo Nieto, Josephine Agyeman Duah, Sonia Deantoni, Brenda Frias Madrid, Fabio Mosca, Brenda Eskenazi, Ann Lambert, Zulfiqar Bhutta, Aris Papageorghiou, Stephen Kennedy, Jose Villar

**Affiliations:** 1 Neonatal Intensive Care Unit, Fondazione IRCCS Ca’ Granda Ospedale Maggiore Policlinico, Milan, Italy; 2 Oxford Maternal & Perinatal Health Institute, Green Templeton College, University of Oxford, Oxford, UK; 3 Center for Environmental Research and Community Health, School of Public Health, University of California, Berkeley, CA USA; 4 Nuffield Department of Women’s & Reproductive Health, University of Oxford, Oxford, UK; 5 Instituto Nacional de Perinatología “Isidro Espinosa de los Reyes”, Mexico City, Mexico; 6 Division of Woman & Child Health, Dept of Paediatrics and Child Health, The Aga Khan University, Karachi, Pakistan; 7 Department of Pediatrics, Federal University of Minas Gerais, Belo Horizonte, Brazil; 8 Division Neonatología, Hospital Materno Infantil Ramón Sarda, Buenos Aires, Argentina; 9 The Aga Khan Hospital, Nairobi, Kenya; 10 Department of Pediatrics, Division of Neonatology, Dokuz Eylul University Faculty of Medicine, Izmir, Turkey; 11 Holy Family Hospital, Brong Ahafo Region, Techiman, Ghana; 12 Azienda Ospedaliera OIRM Sant’Anna Citta della Salute e della Scienza di Torino, Torino, Italy; 13 Neonatology Unit, Hospital Sant Joan de Deú, Barcelona, Spain; 14 Neonatology Department, St. John’s Medical College Hospital, Bangalore, India; 15 Dept of Neonatology, Hôpital Necker-Enfants Malades, Paris, France; 16 General Hospital with Extended Activity Prilep, Trajko Tarcan” bbPrilep, Prilep, Republic of North Macedonia; 17 Department of Clinical Nutrition and Dietetics, College of Health Sciences, University of Sharjah, Sharjah, UAE; 18 Center for Obstetrics, Gynecology and Perinatology, Moscow, Russian Federation; 19 Neonatal Intensive Care Unit of the University of Turin, Turin, Italy; 20 Center for Global Child Health, Hospital for Sick Children, Toronto, ON Canada

## Abstract

**Objective:**

To examine the relationship between etiologically-based preterm birth sub-groups and early postnatal growth according to gestational age at birth.

**Methods:**

Prospective, multinational, cohort study involving 15 hospitals that monitored preterm newborns to hospital discharge. Measures/exposures: maternal demographics; etiologically-based preterm birth sub-groups; very, moderate and late preterm categories, and feeding. Primary outcomes: serial anthropometric measures expressed as *z*-scores of the INTERGROWTH-21^st^ preterm postnatal growth standards.

**Results:**

We included 2320 singletons and 1180 twins: very=24.4% (*n* = 856, including 178 < 28 weeks’ gestation); moderate=16.9% (*n* = 592) and late preterm=58.6% (*n* = 2052). The median (interquartile range) postmenstrual age at the last measure was 37 (36–38) weeks. The ‘no main condition’ sub-group percentage increased from early to late preterm; the ‘perinatal sepsis’ sub-group percentage decreased. ‘Perinatal sepsis’, ‘suspected IUGR’ and ‘fetal distress’ very and late preterm infants had lower postnatal growth patterns than the ‘no main condition’ reference sub-group. This pattern persisted in late but not very preterm infants when postnatal growth was corrected for weight *z*-score at birth.

**Conclusion:**

The proportional contribution of etiologically-based preterm sub-groups and their postnatal growth trajectories vary by preterm category. Postnatal growth is partially independent of fetal growth in the majority of preterm infants (i.e., those born late preterm).

**Impact:**

- Preterm birth, the leading cause of under-5 mortality, is a highly heterogenous syndrome, with surviving infants at risk of suboptimal growth, morbidity, and impaired neurodevelopment.
- Both the proportional contribution of etiologically-based sub-groups and their postnatal growth trajectories vary by preterm category (very/moderate/late).
- The ‘perinatal sepsis’, ‘suspected IUGR’ and ‘fetal distress’ sub-groups amongst very and late preterm infants had lower postnatal growth than the ‘no main condition’ preterm infants. The pattern persisted after adjusting for birth size only in the late preterms.
- Postnatal growth is partially independent of fetal growth in the majority of preterm infants (i.e., those born late preterm).

## Introduction

Preterm birth is the leading cause of under-5 mortality worldwide.^1^ Approximately 15 million babies are born preterm annually and nearly 1 million die from complications of prematurity.^2^ Surviving infants are at increased risk of sub-optimal postnatal growth, short- and long-term morbidity, including impaired neurodevelopment and cardiometabolic disease in adulthood.^3–6^

Preventing preterm birth as well as optimizing postnatal growth, nutrition and development have proven to be a challenge mostly because preterm birth is a highly heterogenous syndrome,^7,8^ associated with a range of etiological factors.^9^ Optimizing postnatal care requires three elements: (a) accurate phenotypic classification of preterm newborns; (b) implementation of evidence-based feeding recommendations, and (c) internationally-derived, standardized measurement of preterm infant growth until 6 months post-term.^10–12^

We have previously described a set of etiologically-based preterm sub-groups with substantial differences in morbidity and developmental outcomes in a multinational cohort of 6529 preterm and term singleton pregnancies (INTERBIO-21^st^ Study) followed from early gestation to 2 years of age.^13^ Here, in a new, large, multinational cohort of preterm neonates, we examine: (1) how the proportional contribution of each preterm birth sub-group changes according to gestational age; (2) the differential patterns of early postnatal growth, controlled for sex, twin pregnancy, mode of delivery and feeding regimens among these etiologically-based sub-groups; and (3) whether these different patterns are similar after controlling for birth measures.

## Methods

The INTERPRACTICE-21^st^ Study was conducted in hospitals or maternity institutions that provided full neonatal care located in 15 cities in 14 countries: Buenos Aires, Argentina; Belo Horizonte, Brazil; Paris, France; Techiman, Ghana; Milan and Turin, Italy; Bangalore, India; Nairobi, Kenya; Prilep, Macedonia; Mexico City, Mexico; Karachi, Pakistan; Moscow, Russia; Barcelona, Spain; Izmir, Turkey; and Oxford, UK. Participating hospitals implemented a standardized package for pregnancy and neonatal evaluation, including the INTERGROWTH-21^st^ pregnancy dating,^14^ newborn size^15,16^ and preterm postnatal growth standards (PPGS),^10^ and promoted evidence-based neonatal and infant feeding recommendations.^17^

From 5 January 2018 to 30 September 2020, we enrolled and followed a cohort of live preterm newborns, born between 23^+0^ and 36^+6^ weeks’ gestation, who were monitored from birth to their hospital discharge. All were born to mothers >18 years of age. Gestational age was estimated by ultrasound measurement of crown-rump length at <14^+0^ weeks’ gestation or head circumference at <24^+0^ weeks’ gestation using INTERGROWTH-21^st^ standards. We oversampled very preterm liveborns (gestational age at birth <32 weeks) across sites after the target sample for individual hospitals was reached to obtain a sufficiently large sample to stratify by sub-group. Neonates were classified by preterm category as: very preterm (23^+0^ to 31^+6^ weeks’ gestation), moderate preterm (32^+0^ to 33^+6^ weeks’ gestation), or late preterm (34^+0^ to 36^+6^ weeks’ gestation).

Standardized forms were used to collect data on maternal demographics, medical and pregnancy-specific conditions, mode of delivery, neonatal and infant anthropometry, feeding practices and comorbidities until hospital discharge. Feeding practices were categorized as parenteral nutrition, tube feeding, oral feeding, and human milk, human milk with fortifiers and various types of formula. All neonatal diagnoses and treatments were standardized,^18^ as were main neonatal care practices using a protocol based on evidence-based practices.^18,19^

Anthropometric measurements were taken using protocols, training materials, and quality control (QC) procedures from the INTERGROWTH-21^st^ Preterm Postnatal Follow-up Study.^20^ Newborn weight, length and head circumference were obtained within 12–24 h of birth using the same equipment at all sites. An electronic scale (Seca, Hamburg, Germany), accurate to the nearest 0.1 g, was used to measure weight at birth and weekly until hospital discharge. An infantometer (Harpenden, Chasmors Ltd, London, UK) was used to measure recumbent length at birth and weekly until hospital discharge. A metallic non-extendable tape (Chasmors Ltd), precise to 1 mm with an 8 cm blank lead-in, was used to measure head circumference at birth and weekly until hospital discharge. All lead anthropometrists were trained to measure newborns according to the study protocol, and, in turn, they trained local staff; all training materials were based on the original World Health Organization protocols. Two trained study anthropometrists independently took all measures twice and compared values using maximum allowable differences of 50 g for weight, 7 mm for length, and 4 mm for head circumference. If any difference exceeded those values, they performed the relevant measurement a third time.

### Statistical analysis

We used a two-step cluster analysis method to identify the preterm sub-groups. Since many births had more than one associated placental, fetal or maternal condition, this method utilized a pre-cluster step to form subclusters with similar newborns and a second step to pool the pre-clusters into the specified number (10) using a hierarchical approach to create clusters as different from each other as possible. The conditions used to cluster newborns were pre-eclampsia, extrauterine infections, chorioamnionitis, perinatal sepsis, early bleeding, mid-late bleeding, suspected intrauterine growth restriction (IUGR, defined as a recorded suspicion of impaired fetal growth during pregnancy based on ultrasound or physical examination), fetal distress, and congenital anomaly. We used between-group linkage as the cluster method and squared Euclidian distance as the interval measure. The 10-cluster model, including the conditions listed above and a ‘no main condition identified’ sub-group, provided categorization consistent with our a priori conceptual classification.^21^

We calculated frequencies (*n*, %) for the distribution of preterm births (*n* = 3500) by sub-group and preterm category. We used chi-squared tests to compare differences in proportions of sub-groups by preterm category, and between singletons and twins. None of these groups had numbers that were less than 10. We determined the percentages of maternal, fetal and placental conditions by preterm birth sub-group. We calculated distributions of maternal characteristics (mean ± SD or *n*, %) and birth outcomes (gestational age, weight, length and head circumference).

To assess postnatal growth, we constructed multi-level mixed effects linear regression models to account for repeated measurements per infant and twin pregnancy. We included infants without congenital anomalies and with at least one anthropometric measure after birth, up to postmenstrual age 42 weeks. We calculated *z*-scores using the INTERGROWTH-21^st^ PPGS.^10^ Each regression model contained one of the relevant growth measures (weight, length or head circumference *z*-score) as the dependent variable, and preterm birth sub-group as the independent variable (using the ‘no main condition’ sub-group as the reference group). We evaluated effect modification between singletons and twins using interaction terms in the models. Since the twin interaction terms were not significant, we included all singletons and twins in all models. We adjusted all models for twin or singleton birth, mode of delivery (cesarean birth), and feeding at follow-up (exclusive breast milk, partial breast milk, formula only or no liquids listed), as determined through detailed questioning of the mothers.

The β coefficients (95% confidence intervals) are provided from models, both unadjusted and adjusted for birth *z*-score, to explore postnatal growth patterns across sub-groups, including and independently of fetal growth. We stratified models by preterm category, calculating a *p*-value for interaction using Wald tests.

The β coefficients (95% confidence intervals) estimated in the analyses can be crudely interpreted as the difference in *z*-scores. More precisely, it is the difference in units of sex adjusted standard deviation (*z*-scores) of the INTERGROWTH-21^st^ PPGS, across the postnatal follow-up period, between a given sub-group as compared with the reference sub-group, adjusted for the variables described above.

The study was approved by the Oxfordshire Research Ethics Committee, institutional research ethics committees at participating sites, and corresponding regional authorities. All parents provided written informed consent.

## Results

We enrolled 3634 preterm newborns distributed across the 15 study sites: 425 (12%), Karachi; 420 (12%), Milan; 399 (11%), Moscow; 375 (10%), Oxford; 338 (9%), Belo Horizonte; 321 (9%), Buenos Aires; 301 (8%), Mexico City; 172 (5%), Nairobi; 163 (4%), Techiman; 161 (4%), Izmir; 136 (4%), Turin; 130 (4%), Barcelona; 104 (3%), Bangalore; 98 (3%), Paris and 91 (3%), Prilep.

From the 3634 preterm newborns, we excluded 130 triplets and four quadruplets, leaving in the analytic sample 3500 newborns (2320 singletons, 1180 twins) from 2923 pregnancies. Of these 3500 newborns, 856 (24.5%) were born very preterm, 592 (16.9%) moderate preterm, and 2052 (58.6%) late preterm, reflecting our oversampling strategy for very preterm newborns. 33.0% of the 856 very preterm newborns were twins, as were 38.2% of the 592 moderate preterm and 32.7% of the 2052 late preterm newborns.

**Fig. 1:**
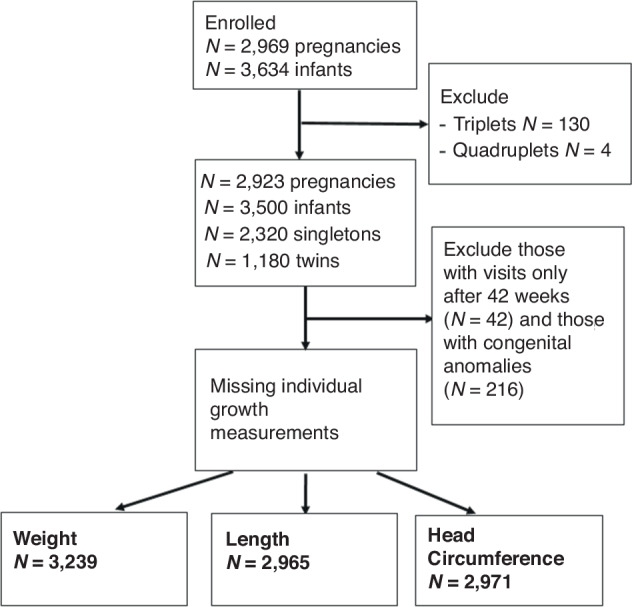
INTERPRACTICE-21^st^ Study participant flowchart.

Of the original 3500 sample, 3239 (92.5%) infants contributed measures of weight (*n* = 3239; singletons = 2144, twins = 1095), length (*n* = 2965; singletons=1945, twins=1020) and head circumference (*n* = 2971, singletons=1950, twins=1021) to the analyses (Fig. 1). The birth characteristics according to preterm birth sub-group and preterm category for all newborns are presented in Table 1.

**Table 1:** Unadjusted birth outcomes indicators (Mean ± SD) according to preterm-birth sub-group for all newborns, in the INTERPRACTICE-21^st^ Study (*n* = 3500)

	No main condition	Pre-eclampsia	Extrauterine Infection	Chorioamnionitis	Perinatal Sepsis	Early Bleeding	Mid-late Bleeding	Suspected IUGR	Fetal Distress	Congenital Anomaly	Very Preterm	Moderate Preterm	Late Preterm
**All infants**	**816**	**408**	**167**	**423**	**346**	**200**	**315**	**282**	**327**	**216**	**856**	**592**	**2,052**
Birth weight (g)	2296 ± 519	2090 ± 594	2244 ± 541	2135 ± 569	1371 ± 595	2032 ± 707	2058 ± 629	1806 ± 492	1878 ± 592	1953 ± 703	1246 ± 369	1839 ± 345	2399 ± 449
Birth length (cm)	44.9 ± 3.3	43.6 ± 3.8	44.3 ± 3.2	43.8 ± 3.5	38.1 ± 5.1	42.9 ± 5.0	43.2 ± 4.6	42.0 ± 3.5	42.0 ± 4.1	41.9 ± 4.8	37.4 ± 3.8	42.3 ± 2.6	45.5 ± 2.6
Birth head circumference (cm)	31.5 ± 2.2	30.9 ± 2.4	31.2 ± 2.3	30.8 ± 2.4	27.2 ± 3.4	30.0 ± 3.5	30.5 ± 3.0	29.9 ± 2.3	29.9 ± 2.8	29.9 ± 3.5	26.6 ± 2.6	30.1 ± 1.7	32.0 ± 1.7
Birth weight for length (kg/cm)	5.07 ± 0.91	4.72 ± 1.05	5.02 ± 0.94	4.81 ± 0.98	3.50 ± 1.10	4.63 ± 1.21	4.67 ± 1.08	4.24 ± 0.89	4.39 ± 1.07	4.54 ± 1.26	3.28 ± 0.73	4.33 ± 0.65	5.25 ± 0.79
Birth weight z-score	−0.21 ± 0.90	−0.34 ± 0.95	−0.13 ± 0.99	−0.02 ± 0.82	−0.55 ± 1.22	−0.23 ± 1.11	0.06 ± 0.84	−1.08 ± 0.99	−0.64 ± 1.04	−0.55 ± 1.40	−0.17 ± 1.17	−0.28 ± 0.93	−0.43 ± 1.02
Birth length z-score	−0.23 ± 1.14	-0.42 ± 1.11	−0.33 ± 1.15	−0.19 ± 0.95	−0.85 ± 1.16	−0.41 ± 1.20	−0.11 ± 1.04	−1.16 ± 1.16	−0.86 ± 1.10	−0.93 ± 1.42	−0.53 ± 1.06	−0.56 ± 1.09	−0.45 ± 1.25
Birth head circumference z-score	0.04 ± 1.07	0.01 ± 1.07	0.01 ± 1.02	0.04 ± 0.96	−0.49 ± 1.17	−0.24 ± 1.17	0.18 ± 0.94	−0.73 ± 1.12	−0.47 ± 1.19	−0.50 ± 1.61	−0.21 ± 1.11	−0.25 ± 1.11	−0.12 ± 1.18
Birth weight for length z-score	−0.19 ± 0.91	−0.28 ± 0.95	−0.07 ± 0.98	−0.03 ± 0.80	−0.33 ± 1.03	−0.19 ± 1.03	0.05 ± 0.85	−0.95 ± 0.92	−0.50 ± 1.03	−0.34 ± 1.33	0.01 ± 0.94	−0.24 ± 0.95	−0.39 ± 1.01
Small for gestational age n (%)	99 (12.1)	59 (14.5)	20 (12.0)	26 (6.2)	89 (25.7)	30 (15.2)	17 (5.4)	131 (46.5)	89 (27.2)	63 (29.2)	138 (16.2)	83 (14.0)	402 (19.6)
Twins n (%)	324 (39.7)	123 (30.2)	66 (39.5)	117 (27.7)	105 (30.4)	67 (33.5)	75 (23.8)	135 (47.9)	99 (30.3)	69 (31.9)	283 (33.1)	226 (38.2)	671 (32.7)

Birthweight (all newborns) ranged from 1371 ± 595 g for infants with ‘perinatal sepsis’ to 2296 ± 519 g for infants with ‘no main condition’; the means ± SD for length at birth were 44.9 ± 3.3, and 38.1 ± 5.1, respectively and for head circumference were 31.5 ± 2.2 cm and 27.2 ± 3.4 cm, respectively for the same sub-groups. Birth size predictably increased from very preterm to late preterm. The rates of small for gestational age (SGA, defined as <10th centile of the INTERGROWTH-21^st^ newborn size standards^16^ or very preterm references^15^) were similar across gestational age categories (very preterm,16.2%; moderate preterm, 14.0%; late preterm,19.6%). The baseline maternal characteristics by preterm birth sub-group and preterm category for all newborns are presented in Supplementary Table 1. Mothers with ‘early bleeding’ were more than twice as likely to have had a previous preterm birth (56%) compared to those with ‘no main condition’ (27%) or ‘pre-eclampsia’ (20%).

### Proportional distribution of the etiologically-based preterm sub-groups according to gestational age at birth

**Fig. 2:**
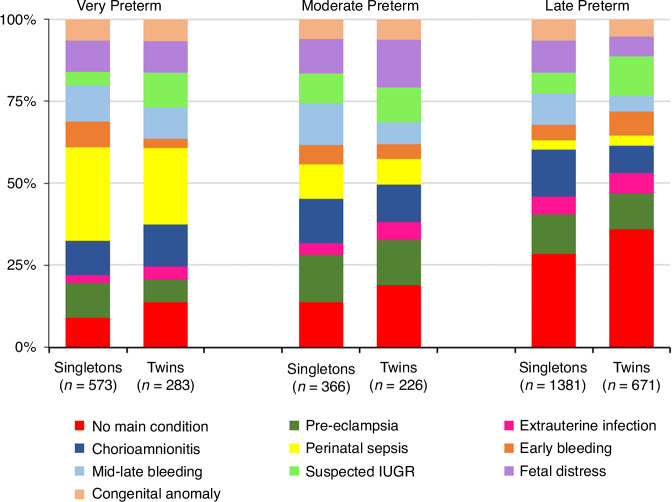
Preterm-birth phenotypes by preterm category comparing singleton and twin pregnancies in the INTERPRACTICE-21^st^ Study (*n* = 3500).

The distribution of the preterm sub-groups according to gestational age category (very, moderate, and late preterm) and for singletons and twins are shown in Fig. 2. The percentage of both singleton and twin newborns with the ‘no main condition’ sub-group increased from very preterm to late preterm; conversely the ‘perinatal sepsis’ sub-group was highest in the very preterm and lowest in the late preterm infants. Twins showed similar patterns to singletons except they had a higher percentage of ‘suspected IUGR’, reflecting the higher rates of SGA at birth.

### Postnatal growth trajectories for weight, length and head circumference across preterm sub-groups

**Fig. 3:**
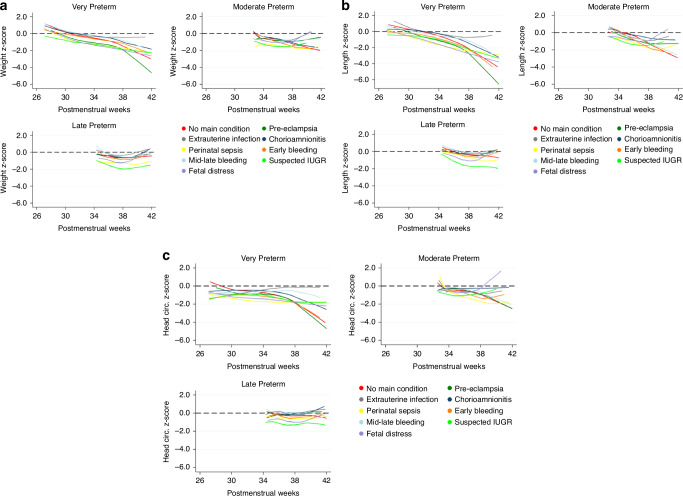
Postnatal infant growth by preterm category in the INTERPRACTICE-21^st^ Study. **a** Postnatal infant weight (z-score) by preterm category in the INTERPRACTICE-21^st^ Study. **b** Postnatal infant length (z-score) by preterm category in the INTERPRACTICE-21^st^ Study. **c** Postnatal infant head circumference (z-score) by preterm category in the INTERPRACTICE-21^st^ Study.

Figure 3a to 3c show postnatal growth trajectories unadjusted for birth measures for weight, length and head circumference by weeks of postmenstrual age expressed as *z*-scores of the PPGS according to preterm category and preterm birth sub-group. Overall, in all preterm infants, the growth trajectory by 38th week for all preterm sub-groups was within −2 (z-score) of the corresponding standards for weight, length and head circumference. After the 38th week the growth trajectory deviated towards −3 and −4 (z score) in the very preterm infant sub-groups, whereas in almost all moderate and late preterm infant sub-groups the growth trend recovered towards zero (z-score) around 42 postconceptional weeks.

### Postnatal growth of weight, length and head circumference by etiologically-based preterm sub-groups with and without adjustment for measures at birth

Table 2 and Fig. 4 a to c present the adjusted beta coefficients (95% confidence intervals) for weight, length and head circumference (*z*-scores of the PPGS) for each preterm sub-group (with the ‘no main condition’ sub-group as the reference group) stratified by preterm category and adjusted for twins, mode of delivery and feeding regimen during follow-up [solid circle] and with adjustment for measures at birth (*z*-score estimated from the size at birth international standards) [open circles].

**Table 2:** Adjusted associations^a,b^ between preterm-birth sub-groups and postnatal anthropometric measures (*z*-scores) according to preterm category in the INTERPRACTICE-21^st^ Study

		Without birth *z*-scores in model^a^			With birth *z*-scores in model^b^		
Preterm birth sub-group	Total	Very Preterm	Moderate Preterm	Late Preterm	Very Preterm	Moderate Preterm	Late Preterm
**Weight z-score**	3239	771	552	1916	771	552	1916
No main condition	809	Ref.	Ref.	Ref.	Ref.	Ref.	Ref.
Pre-eclampsia	405	−0.27 (−0.67, 0.14)	−0.06 (−0.38, 0.27)	−0.21 (−0.37, −0.05)*	0.06 (−0.17, 0.29)	0.19 (0.02, 0.36)*	−0.10 (−0.17, −0.02)*
Extrauterine infection	167	0.24 (−0.35, 0.82)	0.02 (−0.45, 0.49)	0.13 (−0.09, 0.34)	0.34 (0.01, 0.68)*	0.06 (−0.19, 0.31)	0.10 (0.00, 0.20)#
Chorioamnionitis	419	0.32 (−0.06, 0.70)#	−0.14 (−0.47, 0.19)	0.01 (−0.15, 0.17)	0.13 (−0.09, 0.34)	0.08 (−0.10, 0.25)	−0.02 (−0.10, 0.05)
Perinatal sepsis	334	−0.68 (−1.00, −0.36)*	−0.69 (−1.03, −0.35)*	−0.67 (−0.94, −0.40)*	−0.02 (−0.21, 0.16)	−0.15 (−0.33, 0.04)	−0.26 (−0.39, −0.13)*
Early bleeding	193	−0.15 (−0.60, 0.31)	−0.15 (−0.58, 0.27)	−0.10 (−0.32, 0.12)	−0.05 (−0.31, 0.21)	0.18 (−0.04, 0.41)	−0.03 (−0.13, 0.08)
Mid/late bleeding	310	0.48 (0.09, 0.88)*	0.11 (−0.23, 0.46)	0.09 (−0.10, 0.27)	0.14 (−0.09, 0.36)	0.06 (−0.12, 0.24)	−0.06 (−0.15, 0.03)
Suspected IUGR	279	−0.56 (−1.02, −0.10)*	−0.80 (−1.16, −0.45)*	−1.08 (−1.26, −0.89)*	−0.02 (−0.28, 0.24)	0.12 (−0.07, 0.31)	−0.16 (−0.25, −0.07)*
Fetal distress	323	−0.46 (−0.86, −0.06)*	−0.25 (−0.59, 0.08)	−0.67 (−0.85, −0.49)*	0.09 (−0.14, 0.32)	0.15 (−0.03, 0.33)	−0.11 (−0.20, −0.03)*
**Length z-score**	2965	728	517	1720	728	517	1720
No main condition	699	Ref.	Ref.	Ref.	Ref.	Ref.	Ref.
Pre-eclampsia	374	−0.41 (−0.89, 0.08)	−0.21 (−0.62, 0.20)	−0.11 (−0.31, 0.08)	0.08 (−0.23, 0.39)	0.03 (−0.23, 0.28)	−0.02 (−0.12, 0.09)
Extrauterine infection	153	0.20 (−0.53, 0.94)	−0.14 (−0.74, 0.46)	0.07 (−0.19, 0.33)	0.10 (−0.36, 0.56)	0.12 (−0.25, 0.50)	0.14 (0.00, 0.28)*
Chorioamnionitis	395	0.10 (−0.35, 0.55)	−0.18 (−0.61, 0.25)	−0.01 (−0.20, 0.18)	0.01 (−0.27, 0.29)	−0.05 (−0.32, 0.22)	0.03 (−0.08, 0.13)
Perinatal sepsis	295	−1.04 (−1.42, −0.66)*	−0.87 (−1.33, −0.41)*	−0.23 (−0.59, 0.13)	−0.09 (−0.34, 0.15)	−0.35 (−0.63, −0.07)*	−0.08 (−0.28, 0.11)
Early bleeding	184	−0.59 (−1.12, −0.05)*	−0.23 (−0.77, 0.31)	−0.12 (−0.38, 0.14)	−0.11 (−0.45, 0.22)	−0.07 (−0.41, 0.26)	0.02 (−0.12, 0.16)
Mid/late bleeding	297	0.14 (−0.33, 0.60)	−0.14 (−0.58, 0.30)	0.23 (0.01, 0.45)*	0.25 (−0.05, 0.54)#	−0.03 (−0.31, 0.24)	−0.01 (−0.13, 0.11)
Suspected IUGR	277	−0.64 (−1.18, −0.10)*	−1.02 (−1.47, −0.57)*	−1.10 (−1.31, −0.88)*	−0.06 (−0.40, 0.27)	−0.06 (−0.35, 0.22)	−0.22 (−0.34, −0.10)*
Fetal distress	291	−0.83 (−1.32, −0.34)*	−0.45 (−0.88, −0.01)*	−0.67 (−0.89, −0.45)*	0.00 (−0.31, 0.31)	0.13 (−0.15, 0.40)	−0.06 (−0.18, 0.06)
**Head circ. z-score**	2971	730	521	1720	730	521	1720
No main condition	700	Ref.	Ref.	Ref.	Ref.	Ref.	Ref.
Pre-eclampsia	374	-0.16 (−0.61, 0.28)	0.27 (−0.19, 0.72)	−0.09 (−0.30, 0.11)	0.09 (−0.20, 0.39)	0.17 (−0.13, 0.46)	−0.04 (−0.15, 0.07)
Extrauterine infection	153	0.20 (−0.48, 0.88)	0.10 (−0.57, 0.76)	0.15 (−0.12, 0.41)	0.59 (0.14, 1.03)*	0.21 (−0.22, 0.64)	0.12 (−0.03, 0.27)
Chorioamnionitis	397	0.03 (−0.39, 0.44)	0.09 (−0.38, 0.56)	0.04 (−0.16, 0.24)	0.05 (−0.22, 0.32)	0.00 (−0.30, 0.31)	0.13 (0.02, 0.24)*
Perinatal sepsis	297	−0.92 (−1.27, −0.57)*	−0.12 (−0.62, 0.37)	−0.26 (−0.63, 0.11)	−0.14 (−0.37, 0.09)	−0.03 (−0.36, 0.30)	0.09 (−0.11, 0.28)
Early bleeding	184	−0.57 (−1.06, −0.07)*	−0.07 (−0.67, 0.53)	−0.17 (−0.44, 0.10)	0.06 (−0.27, 0.38)	0.09 (−0.30, 0.48)	0.09 (−0.06, 0.24)
Mid/late bleeding	297	0.00 (−0.43, 0.43)	0.27 (−0.22, 0.75)	0.16 (−0.07, 0.39)	0.01 (−0.27, 0.29)	0.08 (−0.24, 0.40)	0.03 (−0.10, 0.16)
Suspected IUGR	277	−0.53 (−1.03, −0.03)*	−0.47 (−0.97, 0.03)#	−0.96 (−1.18, −0.73)*	−0.07 (−0.40, 0.25)	0.17 (−0.16, 0.49)	−0.12 (−0.24, 0.01)#
Fetal distress	292	−0.63 (−1.08, −0.17)*	−0.13 (−0.61, 0.36)	−0.73 (−0.95, −0.50)*	0.08 (−0.21, 0.38)	0.11 (−0.21, 0.42)	−0.05 (−0.18, 0.08)

^a^Models include random intercepts for mother and infant, and adjusted for sex, twin pregnancy, mode of delivery, and feeding at follow-up; visits at post menstrual age ≥42 weeks excluded.

^b^Models include random intercepts for mother and infant, and adjusted for birth z-score, twin or singleton birth, mode of delivery, and feeding at follow-up; visits at post menstrual age ≥42 weeks excluded.

#*p* < 0.1; *p < 0.05.

**Fig. 4:**
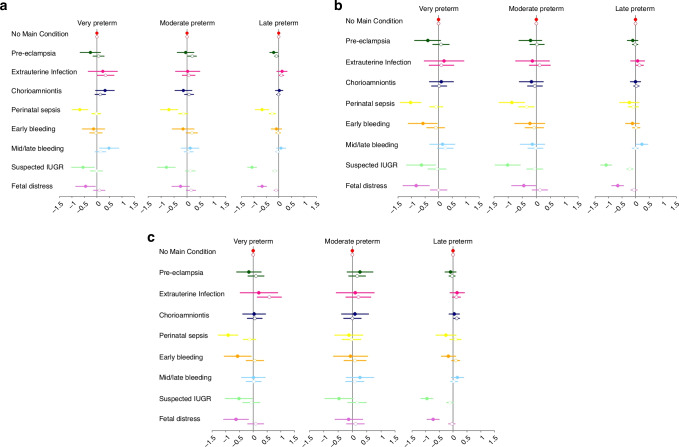
Adjusted associations between preterm-birth phenotypes and postnatal growth according to preterm category for singletons (solid markers) and twins (open markers) in the INTERPRACTICE-21^st^ Study. **a** Adjusted associations^a^ between preterm-birth phenotypes and postnatal weight (*z*-scores) according to preterm category for singletons (solid markers) and twins (open markers) in the INTERPRACTICE-21^st^ Study. ^a^ **b** Adjusted associations^a^ between preterm-birth phenotypes and postnatal length (*z*-scores) according to preterm category for singletons (solid markers) and twins (open markers) in the INTERPRACTICE-21^st^ Study. ^a^ **c** Adjusted associations^a^ between preterm-birth phenotypes and postnatal head circumference (*z*-scores) according to preterm category for singletons (solid markers) and twins (open markers) in the INTERPRACTICE-21^st^ Study. ^a^Models include random intercepts for mother and infant, mode of delivery, and feeding at follow-up; visits ≥42 weeks excluded. Solid markers indicate models for singleton pregnancies; open markers indicate models for twin pregnancies. IUGR intrauterine growth restriction.

#### Very preterm

In the models unadjusted for birth *z*-scores, very preterm infants with the ‘perinatal sepsis’, ‘suspected IUGR’ and ‘fetal distress’ sub-groups had a significantly lower mean postnatal weight compared to those with the ‘no main condition” sub-group (β coefficients ranging from −0.46 to −0.68 of SD). After adjusting for birth weight, such differences were no longer present, implying that the postnatal growth of these preterm infants was mostly related to the differences in fetal growth and birth weight. However, after adjusting for birth weight, the ‘extrauterine infection’ sub-group had a significantly higher mean weight *z*-score (β: 0.34). The ‘perinatal sepsis’, ‘early bleeding’, ‘suspected IUGR’ and ‘fetal distress’ sub-groups had significantly lower mean postnatal length and head circumference (β ranging from: −0.59 to −1.04; and −0.53 to −0.92, respectively). After adjustment for birth measures, these differences became null but the ‘extrauterine infection’ sub-group was now associated with a larger mean head circumference (β: 0.59). However, after controlling for birth measures, the ‘mid-late bleeding’ and ‘extrauterine infection’ sub-groups had significantly higher mean length (β: 0.25) and head circumference (β: 0.59) *z*-scores, respectively than the ‘no main condition’ sub-group.

#### Moderate preterm

In the models unadjusted for birth weight, moderate preterm infants with the ‘perinatal sepsis’ and ‘suspected IUGR’ sub-groups had significantly lower mean weight *z*-scores (β ranging from −0.69 to −0.80); after adjustment for birth weight, these associations became null but the ‘pre-eclampsia’ sub-group had a significantly higher weight *z*-score (β: 0.19). The ‘perinatal sepsis’, ‘suspected IUGR’ and ‘fetal distress’ sub-groups were significantly associated with lower mean length *z*-scores (β ranging from: -0.45 to -1.02) in models unadjusted for birth length; after adjustment for birth length, only the association with ‘perinatal sepsis’ remained (β: -0.35), reflecting differences in postnatal growth for these sub-groups. There were no significant associations with head circumference with or without controlling for birth measures.

#### Late preterm

In the models unadjusted for birth *z*-scores, late preterm infants with the ‘pre-eclampsia’, ‘perinatal sepsis’, ‘suspected IUGR’ and ‘fetal distress’ sub-groups had significantly lower mean weight *z*-scores (β: −0.21 to −1.08); the ‘suspected IUGR’ and ‘fetal distress’ sub-groups had significantly lower mean length (β: −0.67 to −1.1) and head circumference (β: -0.73 to -0.96) *z*-scores. In the models adjusted for birth *z*-scores, the same sub-groups had significantly lower weight z-scores (β: −0.10 to −0.26). The ‘suspected IUGR’ and ‘fetal distress’ sub-groups had significantly lower length (β: −0.67 to −1.10) and head circumference z-scores (β: −0.73 to −0.96); the association with ‘suspected IUGR’ diminished after controlling for birth measures. In contrast, the ‘mid/late bleeding’ sub-group was associated with a higher length *z*-score unadjusted (but not adjusted) for birth length, and the ‘extrauterine infection’ sub-group was associated with higher length *z*-score with adjustment (but not unadjusted) for birth length. Similarly, the ‘chorioamnionitis’ sub-group was associated with a significantly higher (β: 0.13) head circumference *z*-score after adjustment (but not unadjusted) for birth head circumference.

## Discussion

In this large, prospective, multinational study, we have observed: (1) the distribution of etiologically-based preterm birth sub-groups across gestational age at birth; (2) the postnatal growth trajectories for weight, length and head circumference across etiologically-based preterm sub-groups; and (3) the effect of size at birth on the postnatal growth of weight, length and head circumference across etiologically-based preterm sub-groups.

The heterogeneous distribution of etiological sub-groups across gestational ages confirms the complex nature of the preterm birth syndrome, associated with multiple etiological factors that affect pregnancy differently across gestational age at birth.^7,22^ The largest single sub-group, ‘no main condition’, which corresponded to pregnancies without (at the present level of clinical diagnosis) any main maternal, fetal or placental conditions detected, consistent with previous reports,^7,9,13^ increased with gestational age at birth to a high (30%) amongst late preterm newborns. Conversely, the contribution of the ‘perinatal sepsis’ sub-group was greatest amongst the very preterm and lowest in the late preterm newborns. Overall, the three infection-related sub-groups ‘chorioamnionitis’, ‘perinatal sepsis’ and ‘extrauterine infection’ are etiologically associated with 41% of the very preterm newborns. Although they overlap clinically, the prevention and treatment regimens require specific strategies and should be considered in the design and meta-analysis of randomized controlled trials evaluating anti-microbials to prevent preterm birth.

The contribution of the ‘suspected IUGR’ sub-group was higher amongst twins (11%) than singletons (6%), consistent with the evidence that twin pregnancies are more likely affected by lower postnatal growth patterns. This phenomenon could be pathologic (fetal, placental, obstetric, mechanical or underlying maternal causes) or a benign physiological adaptation that optimizes the survival of twin fetuses by decreasing their nutritional demands and uterine overdistention.^23–27^

The postnatal growth trajectories for weight, length and head circumference across etiologically-based sub-groups vary depending on the preterm category. The moderate and late preterm infant sub-groups recovered the growth delay accumulated early in postnatal life, generally by the 42nd post-conceptional week. Conversely, the growth trajectories of very preterm infant sub-groups showed a persistent delay in the period up to and including ‘term’, suggesting that severe prematurity and long hospitalization, often associated with comorbidities, may be responsible for lower postnatal growth patterns and not any particular sub-group. Perhaps for similar reason, the head circumference *z*-scores for very preterm and moderate preterm infants declined in some groups, in particular in the ‘no main condition’ and ‘pre-eclampsia’ sub-groups after 38 postmenstrual weeks.

The effect of size at birth on postnatal growth varied in the different preterm categories and sub-groups, suggesting that intrauterine growth may influence postnatal growth patterns. Postnatal growth of very preterm and late preterm infants with the ‘perinatal sepsis’, ‘suspected IUGR’ and ‘fetal distress’ sub-groups was significantly lower (when growth was not adjusted for birth z-score) than that of infants with the ‘no main condition’ sub-group. This pattern persisted in late preterm but not in very or moderate preterm infants when growth was corrected for *z*-score at birth. Thus, intrauterine growth may influence postnatal growth patterns in both very preterm and late preterm infants. When the model was adjusted for *z*-score at birth, late preterm infants with these sub-groups had a significantly lower weight *z*-score compared to the ‘no main condition’ sub-group, indicating that postnatal growth patterns are partially independent of fetal growth in the great majority of preterm (i.e., late preterm) newborns. Overall, the ‘perinatal sepsis’, ‘suspected IUGR’ and ‘fetal distress’ sub-group infants had significantly lower growth than the ‘no main condition’ sub-group infants.

This study has several unique characteristics including: (1) a very large multinational sample including weight, length, head circumference and weight for length obtained under standardized protocols; (2) participating centers (mostly the leading units in each country or region) were proportionally represented and provided standardized neonatal intensive care unit (NICU) and postnatal care; (3) the gestational ages at birth were evenly distributed, even at low gestational ages due to oversampling this sub-group in the study design; (4) the etiologically-based pregnancy sub-groups were well characterized a priori; (5) infants were monitored throughout their hospital stay and measured daily (weight) or weekly (length, head circumference) using the INTERGROWTH 21^st^ standardized protocols with in-built QC; (6) gestational age at birth was accurately estimated by ultrasound early in pregnancy, and (7) twin pregnancies, etiologically associated with preterm birth, were included and analyzed in sensitivity analyses.

However, the study had some limitations, perhaps the most important being that the follow-up period was short and focused on NICU/hospital stay. The median (interquartile range) postmenstrual age at the last follow-up was 36.7 (35.7–38.1) weeks and the 90th percentile was 42.0 weeks. Hence, infants with a degree of complications are overrepresented in our dataset; so, we stratified all our analyses by preterm category. There is an obvious trade-off between the large sample size of an international collaboration, standardization of equipment and protocols, the promotion of human milk, as the main feeding regimen, and the complexity of large-scale follow-up. We chose to focus on the crucial early neonatal period and on the interaction between pregnancy factors, birth size, human-milk feeding, morbidity and early growth. We acknowledge, however, that a problem with focusing on human feeding is that it is highly associated with the determinants of health and the support mothers receive or do not receive, so it is not a pure exposure that only reflects human milk, but it actually reflects many challenges that mothers face. These factors, that can reflect poverty, social support and economic challenges, should be the focus of future research.

In summary, we have demonstrated that the etiological distribution of preterm birth varies according to gestational age, which should be of relevance for outcome research and preventive strategies at population level; the principal sub-groups were ‘no main condition’ and infectious conditions (chorioamnionitis’, ‘perinatal sepsis’, ‘extrauterine infection’), which should stimulate systematic research to dissect these sub-groups; fetal growth influences postnatal growth for all preterm categories, especially for those with the ‘suspected IUGR’, ‘fetal distress’ and ‘perinatal sepsis’ sub-groups. Hence, there is a need to diagnose reduced extrauterine growth with caution. Finally, preterm infants resulting from singleton and multiple pregnancies share preterm sub-groups, but there are specific patterns in twins that need to be elucidated.

## Supplementary information

Supplementary Table 1

## Data Availability

Anonymized data will be made available upon reasonable request for academic use and within the limitations of the informed consent. Requests must be made to the corresponding author. Every request will be reviewed by the INTERPRACTICE-21^st^ Consortium Executive Committee. After approval, the researcher will need to sign a data access agreement with the INTERPRACTICE-21^st^ Consortium.

## References

[1] Ohuma, E. O. et al. National, regional, and global estimates of preterm birth in 2020, with trends from 2010: a systematic analysis. *Lancet* **402**, 1261–1271 (2023). 37805217 10.1016/S0140-6736(23)00878-4

[2] Liu, L. et al. Global, regional, and national causes of under-5 mortality in 2000-15: an updated systematic analysis with implications for the sustainable development goals. *Lancet* **388**, 3027–3035 (2016). 27839855 10.1016/S0140-6736(16)31593-8 PMC5161777

[3] Eichenwald, E. C. & Stark, A. R. Management and outcomes of very low birth weight. *N. Engl. J. Med.* **358**, 1700–1711 (2008). 18420502 10.1056/NEJMra0707601

[4] Hack, M. et al. Chronic conditions, functional limitations, and special health care needs of school-aged children born with extremely low-birth-weight in the 1990s. *JAMA* **294**, 318–325 (2005). 16030276 10.1001/jama.294.3.318

[5] Järvelin, M. R. et al. Early life factors and blood pressure at age 31 years in the 1966 northern finland birth cohort. *Hypertension* **44**, 838–846 (2004). 15520301 10.1161/01.HYP.0000148304.33869.ee

[6] Sipola-Leppänen, M. et al. Cardiometabolic risk factors in young adults who were born preterm. *Am. J. Epidemiol.* **181**, 861–873 (2015). 25947956 10.1093/aje/kwu443 PMC4445394

[7] Villar, J. et al. Heterogeneity of perinatal outcomes in the preterm delivery syndrome. *Obstet. Gynecol.* **104**, 78–87 (2004). 15229004 10.1097/01.AOG.0000130837.57743.7b

[8] Romero, R. et al. The preterm parturition syndrome. *BJOG* **113**, 17–42 (2006). 17206962 10.1111/j.1471-0528.2006.01120.x PMC7062298

[9] Barros, F. C. et al. The distribution of clinical phenotypes of preterm birth syndrome: implications for prevention. *JAMA Pediatr.* **169**, 220–229 (2015). 25561016 10.1001/jamapediatrics.2014.3040

[10] Villar, J. et al. Postnatal growth standards for preterm infants: the preterm postnatal Follow-up Study of the INTERGROWTH-21^st^ Project. *Lancet Glob. Health* **3**, e681–e691 (2015). 26475015 10.1016/S2214-109X(15)00163-1

[11] de Onis, M., Garza, C., Onyango, A. W. & Martorell, R. Who child growth standards. *Acta Paediatr. Suppl.* **450**, 1–101 (2006).

[12] de Onis, M. & Habicht, J. P. Anthropometric reference data for international use: recommendations from a World Health Organization Expert Committee. *Am. J. Clin. Nutr.* **64**, 650–658 (1996). 8839517 10.1093/ajcn/64.4.650

[13] Villar, J. et al. Association between preterm-birth phenotypes and differential morbidity, growth, and neurodevelopment at Age 2 Years: Results from the INTERBIO-21^st^ Newborn Study. *JAMA Pediatr.* **175**, 483–493 (2021). 33646288 10.1001/jamapediatrics.2020.6087 PMC7922239

[14] Papageorghiou, A. T. et al. International standards for early fetal size and pregnancy dating based on ultrasound measurement of crown-rump length in the first trimester of pregnancy. *Ultrasound Obstet. Gynecol.* **44**, 641–648 (2014). 25044000 10.1002/uog.13448 PMC4286014

[15] Villar, J. et al. INTERGROWTH-21^st^ very preterm size at birth reference charts. *Lancet* **387**, 844–845 (2016). 26898853 10.1016/S0140-6736(16)00384-6

[16] Villar, J. et al. International Standards for Newborn Weight, Length, and Head Circumference by Gestational Age and Sex: The Newborn Cross-Sectional Study of the INTERGROWTH-21^st^ Project. *Lancet* **384**, 857–868 (2014). 25209487 10.1016/S0140-6736(14)60932-6

[17] Cheikh Ismail, L. et al. Preterm feeding recommendations are achievable in large-scale research studies. *BMC Nutr.* **2**, 9 (2016).

[18] Bhutta, Z. et al. Standardisation of neonatal clinical practice. *BJOG* **120**, 56–63 (2013). 23841879 10.1111/1471-0528.12312

[19] Cheikh Ismail, L. et al. Anthropometric standardisation and quality control protocols for the construction of New, International, Fetal and Newborn Growth Standards: The INTERGROWTH-21^st^ Project. *BJOG* **120**, 48–55 (2013). 23841854 10.1111/1471-0528.12127 PMC4019016

[20] Cheikh Ismail, L. et al. Anthropometric protocols for the construction of new international fetal and newborn growth standards: The INTERGROWTH-21^st^ Project. *BJOG* **120**, 42–47 (2013). 23841804 10.1111/1471-0528.12125 PMC4084514

[21] Villar, J. et al. The preterm birth syndrome: a prototype phenotypic classification. *Am. J. Obstet. Gynecol.* **206**, 119–123 (2012). 22177191 10.1016/j.ajog.2011.10.866

[22] Muglia, L. J. & Katz, M. The enigma of spontaneous preterm birth. *N. Engl. J. Med.* **362**, 529–535 (2010). 20147718 10.1056/NEJMra0904308

[23] Townsend, R. & Khalil, A. Fetal growth restriction in twins. *Best. Pr. Res Clin. Obstet. Gynaecol.* **49**, 79–88 (2018). 10.1016/j.bpobgyn.2018.02.004 29661565

[24] Kalafat, E. & Khalil, A. Assessment of fetal growth in twins: which method to use? *Best. Pr. Res Clin. Obstet. Gynaecol.* **84**, 104–114 (2022). 10.1016/j.bpobgyn.2022.08.003 36137872

[25] American College of Obstetricians and Gynecologists Fetal Growth Restriction. **133**, e97-e109 (2019).

[26] Lees, C. C. et al. ISUOG practice guidelines: diagnosis and management of small-for-gestational-age fetus and fetal growth restriction. *Ultrasound Obstet. Gynecol.* **56**, 298–312 (2020). 32738107 10.1002/uog.22134

[27] Society for Maternal-Fetal Medicine, Martins, J. G., Biggio, J. R. & Abuhamad, A. Society for maternal-fetal medicine consult Series #52: diagnosis and management of fetal growth restriction: (Replaces Clinical Guideline Number 3, April 2012). *Am. J. Obstet. Gynecol.* **223**, B2–B17 (2020). 10.1016/j.ajog.2020.05.010 32407785

